# The effect of mindfulness-based stress reduction on resilience of vulnerable women at drop-in centers in the southeast of Iran

**DOI:** 10.1186/s12905-021-01390-6

**Published:** 2021-06-24

**Authors:** Hamideh Adelian, Sedigheh Khodabandeh Shahraki, Sakineh Miri, Jamileh Farokhzadian

**Affiliations:** 1 Health in Disasters and Emergencies Research Center, Institute for Futures Studies in Health, Kerman University of Medical Sciences, Kerman, Iran; 2 Department of Community Health Nursing, Razi Faculty of Nursing and Midwifery, Kerman University of Medical Sciences, Kerman, Iran; 3 Department of Psychiatric Nursing, Razi Faculty of Nursing and Midwifery, Kerman University of Medical Sciences, Kerman, Iran; 4 Nursing Research Center, Kerman University of Medical Sciences, Kerman, Iran

**Keywords:** Mindfulness-based stress reduction, Resilience, Vulnerable women, Drop-in center

## Abstract

**Background:**

Female sex workers and addicted women are among the vulnerable groups who impose high costs on the health system of every society. They are prone to psychological problems such as anxiety, stress, and reduced resilience due to their lifestyles. Since mindfulness-based stress reduction (MBSR) has been applied frequently by many psychotherapists to treat stress and anxiety, the present study investigated the effectiveness of MBSR on resilience of the vulnerable women.

**Methods:**

The statistical population of this quasi-experimental study consisted of all eligible women who referred to the drop-in centers in Kerman in the southeast of Iran. Followed by random sampling, 63 vulnerable women were randomly assigned into the intervention (n = 30) and control (n = 33) groups. The MBSR intervention was conducted for the intervention group in eight 90-min sessions. Demographic information questionnaire and Connor-Davidson resilience scale were administered to collect data prior to and one month after the MBSR intervention.

**Results:**

The pre-test resilience score was not significantly different between the intervention (53.40 ± 10.49) and the control (54.5 ± 9.27) groups (t = 0.43, *p* = 0.66). However, the posttest resilience score in the intervention group (60.66 ± 6.71) was significantly higher than the control group (53.88 ± 7.54) (t = 3.58, *P* = 0.001). Moreover, a comparison between the pretest and posttest scores revealed a significant decrease in resilience scores of the control group (t = 2.81, *p* = 0.009).

**Conclusion:**

Since MBSR intervention increased resilience of the vulnerable women in the intervention group, researchers, managers, counselors, community health nurses, and psychiatric nurses are suggested to implement related interventions to promote the health of women, especially vulnerable ones.

**Supplementary Information:**

The online version contains supplementary material available at 10.1186/s12905-021-01390-6.

## Background

Although the health status of every communities is associated with the health of women indirectly, women are more prone to psychological damage due to their roles as mothers, wives, and society members [1]. Complicated social relationships, poor emotional relationships in families, and increase of illegal immigrations to large cities and suburbanization are among the factors that pave the way for the spread of social deviances, including prostitution and sex work, especially among women. Such a condition threatens the socioeconomic and mental health of families and communities [2]. The prevalence of sex work among over-15-year-old girls was estimated as 0.4–3.4 in African countries, 0.2–2.6 in Asian countries, and 0.1–1.4 in European countries. Although no exact and formal statistics were published on the rate of sex work in Iran, 4,000–30,000 street-walkers and 200,000–300,000 female sex workers were estimated [3]. Female sex workers, addicts, and wives of imprisoned men are considered as examples of vulnerable and risky women [2, 4]. Vulnerable women are not only deprived of emotional support, but also exposed to violence, humiliation, stigma and various physical, as well as mental and verbal discriminations in families and society [5]. Thus, they have been marginalized and stigmatized in the society [6]. Wechsberg et al. concluded that addicted women were more vulnerable and could not protect themselves sexually [7]. As a result of these unpleasant experiences, anxiety is caused in vulnerable women [8].

Anxiety is a physiological manifestation of stress, which is actually an unpleasant emotional experience leading to increased activity of the autonomic nervous system [9]. Given that women are more sensitive to stressful experiences, they suffer from anxiety and stress-related psychiatric disorders twice more than men. In addition, men and women are different in neural circuits that affect emotional responses [10]. Resilience is one of the protective factors against stress [11]. While some people are very vulnerable to life problems, some others cope with problems calmly and logically; the difference between the two groups rests in their level of resilience. Resilience is defined as a flexible response to the life stressors [12]. According to the theory of Lazarus and Folkman, “stress is an interaction between the person and the environment; whereby, individuals appraise environmental demands as outweighing their abilities to meet those demands” [13]. Resilience is the capacity to recover quickly from difficulties and return to the previous level of function. In other words, resilience is the ability to cope with a crisis mentally or emotionally and to return to the pre-crisis status quickly [3]. Windle (2011) defined resilience as the ability to negotiate, adapt to, or manage considerable sources of stress or trauma. Individuals' access to assets and resources, life styles, and environments facilitate this capacity for adaptation and ‘bouncing back’ in the face of adversity. However, resilience is experienced differently during the life [14].

Resilience is influenced by health status, that is to say, chronic adversity or psychopathology may interfere with a person's ability to be ‘resilient’. As a result, mentally ill patients have lower levels of resilience than the general population. Resilience can be improved through treatment, so that greater levels of resilience are associated with higher levels of global improvement. The growing focus on health promotion and well**-**being and transition from pathology and problem-orientation has provided an opportunity to reform the role of resilience in health [15].

Vulnerable women such as female sex workers have lower levels of resilience pertaining to sociological causes such as gender, socioeconomic status, poverty, and substance abuse [16]. A qualitative study on the resilience of sex workers found that homelessness, drug use, and experience of sexual abuse impact resilience negatively. However, resilience can be improved by protective factors such as education, social support, access to health care [17], and active coping styles [18].

Vulnerable women should be trained to increase their resilience in the face of stressful situations and react appropriately [19]. Mindfulness, as one of the methods of stress management, has recently attracted much attention [20, 21]. Stress management skills such as mindfulness-based stress reduction (MBSR) reduce stress and its unpleasant consequences. These skills facilitate coping with stress via coordinated behaviors to meet the environmental needs [22]. In the case of appropriate implementation of mindfulness, it can complement traditional cognitive-behavioral interventions, increase their effectiveness, and prevent recurrence of stress-related problems [23].

Mindfulness-Based Stress Reduction (MBSR) was invented by Kabat-Zinn at the Stress Reduction Clinic at the University of Massachusetts Medical Center. Mindfulness is defined as purposeful and non-judgmental consideration of the present moment to the unfolding of experience moment to moment. By cultivating greater awareness of mind and body, MBSR attempts to empower individuals to control and ease their stress [24, 25]. In this treatment, mindfulness skills are thought for coping with life stresses and raising awareness from the present time that include meditations related to thought, calmness, and Hatha yoga. The MBSR involves paying attention to the present time in a special and targeted way without judgment. One of the main goals of this program is health promotion and stress reduction, increasing mindfulness skill and self-acceptance in individuals. Application of Hatha yoga practices in this treatment causes states of relaxation, breath awareness, and body sensations in individuals, which leads to a kind of unity between body and mind [26].

Mindfulness teaches us how to consider our issues, including awareness of breathing, negative thoughts and worries about the future, re-concentration on the present time, and consideration of every thought entering our mind without judgment [27]. Conscious attention helps people to get rid of their useless habits and reactions and to reach more useful and appropriate responses [28]. When individuals respond to thoughts and somatic feelings, they become aware of negative emotions such as anxiety; so, they can improve their coping skills by focusing on the source of stress during the mindfulness sessions [29]. Several hypotheses explained mechanisms of mental concentration in mindfulness, one of which mentions that exposure to or tendency to experience difficult emotions (such as anxiety, stress, anger), awareness, and observation of these emotions allow people to better cope with and regulate difficult emotions. Another hypothesis is that awareness of thoughts and somatic feelings helps people to cope with stress and increase their resilience [30].

Researchers considered mindfulness as one of the interventions to increase resilience [31–33]. Moreover, numerous studies have examined the effectiveness of mindfulness-based interventions on the psychological variables of vulnerable groups. For example, Sancho et al. (2018) in a systematic review showed that mindfulness-based interventions were effective in the treatment of substance and behavioral addictions. They suggested that future studies should be conducted on young and vulnerable populations with longer follow-ups [34]. Other studies also confirmed the effect of the MBSR on participants' resilience in vulnerable groups [28, 35–39].

Owing to the fact that vulnerable women such as female sex workers experience great stress due to social harms, which lead to their psychological social problems and decrease their resilience, it is necessary to conduct mental health interventions among them. Although MBSR contains valuable trainings with regard to interpersonal issues, limited research was conducted on this vulnerable group. This study aimed to investigate the effect of MBSR on resilience of vulnerable women who referred to the drop-in centers. The study hypothesis was: vulnerable women who attend an eight-week MBSR intervention demonstrate higher levels of resilience and its subscales, compared to the control group.

## Methods

### Study design and settings

The present quasi-experimental study was carried out among the intervention and control groups with the pretest and posttest design to examine the effect of MBSR training on resilience of vulnerable women. The research settings included two drop-in centers, one affiliated to Kerman University of Medical Sciences and the other affiliated to the Welfare Organization located in Kerman, the largest city in southeastern Iran. Establishment of a drop-in center (DIC) can provide health services, reduce harm, and increase mental health of vulnerable women. These centers were established in more than 38 densely populated areas of Iran, especially by universities of medical sciences and non-governmental organizations affiliated with the Welfare Organization [40]. These centers are the primary source of assistance for women who have left home. They help clients to meet their health needs such as counseling, mental health, behavioral care and midwifery examinations, health education and promotion, shelter, and food. The drop-in centers are aimed to reduce the socioeconomic and health consequences and substance abuse, etc. Women who use these centers are less exposed to sexual victimization or criminal risks such as drug trafficking [41].

### Study population and sampling

The statistical population in this study included all vulnerable women who referred to drop-in centers in Kerman during the data collection (N = 140). The center No. 1 affiliated to the welfare organization had 40 clients and center No. 2 affiliated to the University of Medical Sciences had 100 clients. The sample size was calculated as 60 using the sample size formula, but 70 participants were included in the study followed by taking into account 15% dropout probability and the increase in the study power. To facilitate access to participants and receive higher cooperation, the intervention group members were selected from center No. 1 and the control group was selected from the center No. 2. To collect the participants, a list of clients was prepared from both centers and 35 people were selected from each center using a random number table. As a result, 70 women were selected from both centers. It should be noted that both centers had the same conditions and provided similar services to the clients.

Inclusion criteria were having no severe mental and physical disorders, no severe learning disabilities, sufficient time for mindfulness practices, 18 years of age and older, and no history of participation in MBSR sessions or similar interventions. Exclusion criteria included the participant's unwillingness to continue MBSR sessions, absenteeism in more than two sessions, as well as experience of significant psychological and emotional changes during the study, such as the death of a family member.

### Measurements

To collect data, demographic information questionnaire was administered on the participants' occupation, age, marital status, education, history of being imprisoned, family support, friends’ support, insurance coverage, number of children, and type of vehicle used. The questionnaire developed for this study is provided as Additional file 1.

Furthermore, the Persian version of Connor-Davidson Resilience Scale (CD-RISC) was used to assess the level of resilience. The CD-RISC comprises of 25 items, each rated on a five-point scale (0–4), so that higher scores reflect greater resilience (Additional file 2). The reliability of the original CD-RISC-25 was confirmed via test–retest correlation (r = 0.87) and its internal consistency (α = 0.89) was assessed in the studies conducted on American participants. Its convergent validity showed that the scale had positive correlations with psychological variables such as hardiness and social support, but negative correlations with perceived stress, degree of disability, and stress vulnerability [15]. Iranian researchers determined psychometric properties of CD-RISC-25 by receiving permission from its original authors and reported the Cronbach's alpha of 0.94 for its reliability. Exploratory factor analysis and principal factor analysis were used to corroborate its validity [42].

### Data collection and intervention procedure

The present study was conducted from the November until February of 2020. At the beginning of the study, the participants completed demographic information questionnaire and CD-RISC. Moreover, the intervention and control groups recompleted the questionnaires one month after the last session of MBSR simultaneously. To increase the participation opportunity in MBSR, the participants in the intervention group were divided into two groups of 17 to 18. The participants were required to receive MBSR in 90-min sessions for eight weeks. To ensure low attrition rate, the researcher and administrators of the DIC set the intervention time based on the participants' convenience. The training sessions were held with the same training protocol twice a week at 10–11:30 am when the participants attended the center for routine services. Participants were required to choose a regular time and day to attend the course throughout the week, but when this was not possible, they could attend an alternative session within the same week. Participants were also encouraged to do practical exercises including meditations and other mindfulness practices, such as a mindful walk, mindful eating, and habit breakers at the center hall. The recommended exercises varied throughout the course ranging from eight min to about 15–25 min per day. They were also given the opportunity to talk with the psychologist in the case that they had any problems or needed clarifications. Participants were followed by the first researcher in terms of their reliance to the practical exercises and to check whether they had a negative experience with MBSR. No adverse effect was observed during the MBSR in the intervention group, only five participants did not complete the MBSR sessions.

A clinical psychologist professional in MBSR prepared the MBSR intervention package, and trained it. Detailed description of MBSR sessions is shown in Table 1. The participants in the control group received the routine care such as counseling, mental health, behavioral care, midwifery examinations, health education, promotion, shelter, and food. No training was provided for the control group during the study.

**Table 1:** The MBSR Protocol

Session 1	Short introduction to the program, explanations about confidentiality and privacy of the information, asking participants to introduce themselves, eating practices (introduction to mindfulness meditation)
Session 2	Body scan meditation, discussions about barriers to exercise, introduction of seated meditation with awareness about breathing, breath-work, sitting, yoga, and eating meditation
Session 3	Seated meditation with a focus on breathing and body, calendar of unpleasant events
Session 4	Seated meditation with focusing on breathing, body senses, sounds and thoughts, group exploration, conscious walking
Session 5	Reacting and responding to stress, guided seated meditation, discussing people's observations of reacting to stressful events during the week, discussions about life-changing events and their relationships with health, discussing about mindful walking, finishing classes with a seated meditation, gentle stretching, group discussion
Session 6	Practicing alternative moods, thoughts and perspectives, short seated meditation, sharing feelings for 30 min
Session 7	Four-dimensional meditation, a discussion of the pleasant and unpleasant life events
Session 8	Body scan meditation, breathing exercises, implementation of the trainings

### Statistical analysis

For data analysis, SPSS _21_ was run using descriptive (frequency, percentage, mean and standard deviation) and inferential statistics (independent samples *t*-test, paired *t*-test, chi-square test, multivariate linear regression, and the analysis of covariance) at the significance level of ≤ 0.05.

## Results

### Demographic information

Of 70 participants, members of the intervention (n = 30) and control (n = 30) groups completed the questionnaires. The study was completed by a 90% response rate since five individuals from the intervention group did not complete the MBSR sessions and the post-intervention questionnaires; so, they were excluded from the study. Moreover, two individuals from the control group were excluded because of incomplete questionnaires.

The results showed that most women in the intervention and control groups were housewife (83.3%, 87.9%) and married (36.7%, 63.6%) with elementary level of education (40.00%, 33.3%). Considering the participants' age, 41.4% of women in the intervention group were 40–50 years old and 59.4% of the participants in the control group were 30–40 years. Table 2 represents additional demographic information of the participants in the intervention and control groups. Furthermore, no differences were observed in the baseline measures of demographic variables except for marital status.

**Table 2:** Comparison of demographic information of intervention and control groups

Variables	Groups	Intervention group		Control group		*χ2*	*p*
	Categories	n	%	n	%		
Occupation	Housewife	26	86.70	29	87.90	5.9	0.20
	Employed	4	13.30	1	3.00	6	
	Self-employed	0	0	1	3.00		
	Retired	0	0	2	6.10		
Age groups	20–30	2	6.60	3	9.00	2.9	0.40
	31–40	11	36.60	19	57.80	3	
	41–50	13	43.30	8	24.20		
	> 50	4	13.50	3	9.00		
Family support	Yes	13	43.30	21	63.60	2.6	0.11
	No	17	56.70	12	36.40	0	
Friends’ support	Yes	10	33.30	11	33.30	00	
	No	20	66.70	22	66.70		
Education level	Elementary	12	40.00	11	33.30	3.1	0.37
	High school	10	33.30	9	27.30	5	
	Diploma	8	26.70	13	39.40		
Marital status	Single	0	0	3	9.10	96	**0.01**
	Married	11	36.70	21	63.60	9	
	Widowed	8	26.60	5	15.20		
	Divorced	11	36.70	4	12.10		
History of being imprisoned	Yes	11	36.70	6	18.20	3.4	0.18
	No	19	63.30	27	81.80	2	
Children No	0	2	6.08	7	21.90	2.7	0.25
	1	8	26.6	7	21.90	3	
	≥ 2	20	66.6	19	56.20		
Type of vehicle	Personal vehicle	1	3.30	6	18.20	3.5	0.17
	Public	22	73.30	21	63.60	3	
	Transportation						
	None	7	23.30	6	18.20		
Insurance coverage	Social security	9	30.00	8	24.20	4.6	0.46
	Relief foundation	3	10.00	0	0	4	
	Rural	4	13.30	4	12.10		
	Health	6	20.00	6	18.10		
	Therapeutic services	1	3.40	2	6.30		
	None	7	23.30	13	39.30		
Under physical or psychological violence of the family	Yes	20	66.7	24	72.70	0.2	0.60
	No	10	33.33	9	27.30	7	
Housing situation	Personal house	12	40.00	9	27.20	1	0.60
	Rented house	18	60.00	24	72.80		
Residential area	City	27	90.00	23	69.70	3.8	0.14
	Suburbs	2	6.60	8	24.20	7	
	Village	1	3.4	2	6.10		

Bold *p*-values are significant at level of ≤ 0.05

### Resilience

Table 3 shows the level of resilience and its dimensions in the study groups before and after the intervention. The resilience total score in the intervention group increased statistically at the posttest (t = − 7.81, *p* = 0.001). A comparison of the pretest and posttest scores in the control group revealed a significant decrease in resilience (t = 2.81, *p* = 0.009).

**Table 3:** Comparison of scores of resilience between the control and intervention groups before and after the MBSR program

Variable	Time	Pretest	Posttest	Mean difference	Paired-t test	*P-* value
	Groups	Mean ± SD	Mean ± SD			
Personal competence, high standards, and tenacity	Intervention	16.36 ± 4.84	20.76 ± 2.86	4.4	8.93	**0.001**
	Control	16.60 ± 5.40	16.35 ± 4.49	− 0.25	2.7	**0.01**
	Independent t-test	0.184	4.49			
	*P*- value	0.85	**0.001**			
Trust in one’s instincts, tolerance of negative affect, and strengthening of the effects of stress	Intervention	11.63 ± 4.55	14.26 ± 3.07	2.63	7.18	**0.001**
	Control	11.93 ± 4.50	11.27 ± 4.05	− 0.66	1.69	0.1
	Independent t-test	0.26	3.2			
	*P-* value	0.79	**0.002**			
The positive acceptance of change, and secure relationships	Intervention	10.03 ± 1.32	12.23 ± 2.40	2.2	6.33	**0.001**
	Control	12.42 ± 2.19	11.96 ± 2.05	− 0.46	1	0.32
	Independent t-test	0.33	− 4.32			
	*P*- value	0.74	**0.001**			
Control	Intervention	6.60 ± 2.63	8.20 ± 1.66	1.6	5.44	**0.001**
	Control	6.96 ± 2.68	7.10 ± 2.33	0.14	0.13	0.89
	Independent t-test	0.55	2.06			
	*P*- value	0.58	**0.04**			
Spiritual influences	Intervention	6.56 ± 1.25	7.40 ± 0.77	0.84	7.81	**0.001**
	Control	6.81 ± 1.26	6.60 ± 1.16	− 0.21	2.11	**0.04**
	Independent t-test	− 0.79	3.14			
	*P*- value	0.43	**0.003**			
Resilience total	Intervention	53.40 ± 10.49	60.66 ± 6.71	7.26	− 7.81	**0.001**
	Control	54.50 ± 9.27	53.88 ± 7.54	− 0.62	2.81	**0.009**
	Independent t-test	− 0.43	3.58			
	*P*- value	0.66	**0.001**			

Bold *p*-values are significant at level of ≤ 0.05

In the pretest, no statistically significant difference was found in the scores of resilience between the intervention (53.40 ± 10.49) and control (54.5 ± 9.27) groups (t = 0.43, *p* = 0.66). In the posttest, a statistically significant difference was observed between the intervention (60.66 ± 6.71) and control (53.88 ± 7.54) groups in terms of the total score of the resilience (*t* = 3.58, *p* = 0.001).

Covariance analysis test was run to confirm the results of Table 3, showing that by controlling the impact of pretest and marital status on the resilience, significant differences were found between the control and intervention groups with regard to the total posttest scores of resilience and its dimensions (Table 4).

**Table 4:** Summary of covariance analysis for the two groups of control and intervention

Variable		df	Mean square	F	P- value
*Personal competence, high standards, and tenacity*					
Corrected model		4	222.53	68.07	** < 0.001**
Intercept		1	75.66	23.14	** < 0.001**
Pre test		1	0.012	0.004	0.952
Marital status		1	6.09	1.86	0.178
Time		1	593.6	181.59	** < 0.001**
Group		1	270.78	82.83	** < 0.001**
Error		55	3.26		
*Trust in one’s instincts, tolerance of negative affect, and strengthening of the effects of stress*					
Corrected model		4	188.72	90.91	** < 0.001**
Intercept		1	101.67	48.97	** < 0.001**
Pre test		1	6.59	3.17	**0.08**
Marital status		1	8.59	4.13	**0.04**
Time		1	606.67	292.24	**< 0.001**
Group		1	163.29	78.66	** < 0.001**
Error		55	2.07		
*The positive acceptance of change, and secure relationships*					
Corrected model		4	35.06	21.48	** < 0.001**
Intercept		1	21.39	13.10	** < 0.001**
Pre test		1	0.097	0.06	0.80
Marital status		1	4.81	2.95	0.09
Time		1	66.93	41.01	**< 0.001**
Group		1	58.73	35.99	** < 0.001**
Error		55	1.63		
*Control*					
Corrected model		4	43.56	32.73	** < 0.001**
Intercept		1	17.32	13.02	**< 0.001**
Pre test		1	0.84	0.63	0.43
Marital status		1	0.200	0.15	0.70
Time		1	153.41	115.28	** < 0.001**
Group		1	18.97	14.26	** < 0.001**
Error		55	1.33		
*Spiritual influences*					
Corrected model		4	11.28	29.71	** < 0.001**
Intercept		1	11.28	29.72	** < 0.001**
Pre test		1	0.93	2.47	0.12
Marital status		1	0.44	1.16	0.28
Time		1	32.51	85.63	**< 0.001**
Group		1	11.35	29.90	** < 0.001**
Error		55	0.38	69.41	
*Resilience total*					
Corrected model		4	725.19	54.54	** < 0.001**
Intercept		1	569.79	0.99	** < 0.001**
Pre test		1	10.40	0.38	0.32
Marital status		1	3.99	210.15	0.54
Time		1	2195.50	79.60	** < 0.001**
Group		1	831.6		** < 0.001**
Error		55	10.44		

Bold *p*-values are significant at level of ≤ 0.05

In addition, to verify the impact of age groups and education level on the resilience process, the multivariate linear regression (Forward stepwise method) was conducted. The results showed that these variables were not significant predictors of resilience in women (Table 5).

**Table 5:** Multivariate regression model for variables age groups, education level, and resilience

	Unstandardized coefficients		standardized coefficients		
	B	Standard error	B	t	*p*-value
Constant	57.15	7.13		8	** < 0.001**
Age groups	1.63	1.23	0.15	1.61	0.201
Marital status	− 1.69	1.19	− 0.18	− 1.42	0.160
Education level	− 1.03	1	− 0.11	− 1.02	0.309
Time	0.063	1.01	0.008	0.06	0.951
Group	− 5.91	1.9	− 0.38	− 3.11	**0.003**

Bold *p*-values are significant at level of ≤ 0.05

## Discussion

The present study examined the effect of MBSR on the resilience of vulnerable women. The findings supported the research hypothesis showing that resilience scores of the intervention group increased significantly at the posttest. Consistent with the current finding, previous studies reported higher posttest resilience scores in participants of the intervention group after and even during the follow-up stages of the MBSR intervention [32, 36, 37]. Numerous studies in Iran confirmed the effect of mindfulness-based interventions on the resilience of vulnerable populations, including the divorced mothers [35], patients with systemic lupus erythematosus [38], mothers of children with cancer [43], and war veterans [44]. Similar results were also reported in studies on the wives of men with schizophrenia [39], female victims of domestic violence [45], wives of veterans with psychiatric diseases [46], female-headed households [28], mothers of children with learning disabilities [47], and husband’s infidelity [48]. Such similarity in the findings may be due to the application of an intervention with similar protocol on vulnerable groups. Vulnerable groups are often neglected in the society and receive little training in this field. Such interventional programs can motivate and encourage them to learn and improve the level of resilience. Our findings suggested that in spite of critical factors and high pressures, resilient individuals can improve their social capacities and overcome problems. Therefore, vulnerable people should be trained to increase their resilience and cope with challenging situations with greater bio-psychological and social balance.

In contrast, Dyrbye et al. (2017) observed no change in resilience of medical students after stress management and resilience training courss [49]. Ünbül and Güneri study showed a direct and poor correlation between mindfulness and resilience in high school students with poor socioeconomic conditions [50]. The discrepancy in the findings can be attributed to the difference in study design and setting, data collection tools, and participants' demographic variables (gender, age, as well as cultural and geographical status). For example, Dyrbye et al. conducted a quasi-experimental study without a control group on medical students who were under great stress at Mayo Clinic School of Medicine [49]. The study of Ünbül and Güneri was a correlational research on underprivileged adolescents’ resilience and mindfulness in Turky [50].

Our findings showed that the mean posttest resilience score in the control group was significantly lower than the pretest score. This can be justified by mentioning that post-intervention evaluation coincided with the outbreak of COVID-19, which has affected the level of communication with friends, family, and healthcare providers. Low level of communication reduced social support and the economic conditions resulting from quarantine reduced the rate of resilience. In agreement with our results, several studies reported that the resilience score in the control group decreased after the MBSR intervention [44, 45, 48]. In addition, some studies reported that the resilience score of women in the control group increased after the intervention [39, 47]. The findings of this study highlight the importance of initiative approaches to promote resilience of the vulnerable women. Vulnerable women experience many stressful situations during their lives. Inability to cope with and manage these situations can influence vulnerable women, reduce their general health, and increase their psychological problems. Therefore, MBSR and other psychological interventions are suggested to increase the women's resilience and coping skills in stressful situations and help them to better control their life events and face the challenging situations.

## Limitations

The present study has some limitations. First, two different centers were used for allocating the participants into the intervention and control groups to prevent treatment diffusion that could endanger the internal validity of the study and influence behavior of the control group. To meet this methodological limitation, the researchers tried to match but keep the study groups apart from each other at the beginning of the study [51]. However, the significant difference between two groups in terms of marital status indicated a selection bias despite randomization. This limitation was controlled using covariance analysis. Second, this study was conducted only in two DICs in southeastern Iran with a small sample size and in two measurement steps. Therefore, we recommend future researchers to conduct similar studies in other settings with larger sample sizes and to examine the effects of MBSR intervention over several follow-ups, such as 2–6 months. A follow-up study is also recommended to assess the effectiveness of the MBSR training as well as the possibility of verifying temporal aspects in resilience. Finally, since no specific tool was found to assess the resilience of vulnerable women, further studies are suggested using a special, valid, and reliable tool to assess resilience of vulnerable women.

## Conclusions

The findings showed that MBSR intervention significantly increased the resilience of vulnerable women. Therefore, counselors, community health nurses, and psychiatric nurses are recommended to plan and implement interventional courses to promote the health of women, especially the vulnerable groups. Moreover, we suggest community health and psychiatric nurses to develop their knowledge and skills about MBSR intervention. Consequently, nurse managers, decision-makers, and nurse educators should hire well-competent and efficient nurses in providing psychological intervention such as MBSR. It is essential to strengthen the interaction and collaboration of nurse educators with psychologists and counselors in nursing schools in to develop such competencies in future nurses. Researchers should also examine the effect of MBSR on other psychological variables such as anxiety, stress, and social adjustment of vulnerable groups in different cultures and contexts.

## Supplementary Information

**Additional file 1**: Demographic information questionnaire.

**Additional file 2**: Connor-Davidson Resilience scale.

## Data Availability

The data are available upon request to the corresponding author after signing appropriate documents in line with ethical application and the decision of the Ethics Committee.

## Abbreviations

CD-RISC: Connor-Davidson Resilience scale
MBSR: Mindfulness-based stress reduction
DIC: Drop-in center
