# Supplementary material for: The effect of mindfulness-based stress reduction on resilience of vulnerable women at drop-in centers in the southeast of Iran
Source: BMC Womens Health. 2021 Jun 24;21:255. doi: 10.1186/s12905-021-01390-6 (PMC8222952; doi:10.1186/s12905-021-01390-6)
Supplement: Supplementary file 1 — Additional file 1: Demographic information questionnaire. [file 12905_2021_1390_MOESM1_ESM.docx]

**Demographic information questionnaire**

| **Occupation** | Housewife □ Employed □ Self-employed □ Retired □ |
| --- | --- |
| **Age groups** | 20-30 □ 31-40 □ 41-50 □ >50 □ |
| **Family support** | Yes □ No □ |
| **Friends’ support** | Yes □ No □ |
| **Education level** | Elementary □ High school □ Diploma □ |
| **Marital status** | Single □ Married □ Widowed □ divorced □ |
| **History of being imprisoned** | Yes □ No□ |
| **Children No.** | 0 □ 1 □ ≥2□ |
| **Type of vehicle** | Personal vehicle □ Public Transportation □ None □ |
| **Insurance coverage** | Social security □ Relief foundation □ Rural □ Health □ Therapeutic services □ None □ |
| **Under physical or psychological violence of the family** | Yes □ No □ |
| **Housing situation** | Personal house □ Rented house □ |
| **Residential area** | City □ Suburbs □ Village □ |
