# Supplementary material for: The effect of mindfulness-based stress reduction on resilience of vulnerable women at drop-in centers in the southeast of Iran
Source: BMC Womens Health. 2021 Jun 24;21:255. doi: 10.1186/s12905-021-01390-6 (PMC8222952; doi:10.1186/s12905-021-01390-6)
Supplement: Supplementary file 2 — Additional file 2: Connor-Davidson Resilience scale. [file 12905_2021_1390_MOESM2_ESM.docx]

**Connor-Davidson Resilience scale (CD-RISC)**

CD-RISC has five subscales:

1. Personal competence, high standards, and tenacity (items 10, 11, 12, 16, 17, 23, 24, 25(;

2. Trust in one’s instincts, tolerance of negative affect, and strengthening of the effects of stress (items 6, 7, 14. 15, 18, 19, 20);

3. The positive acceptance of change, and secure relationships. (Items 1, 2, 4, 5, 8);

4. Control (items 13, 21, and 22);

5. Spiritual influences (items 3, 9).

All items of the CD-RISC are evaluated on a 5-point Likert scale ranging from 0-4: not true at all (0), rarely true (1), sometimes true (2), often true (3), and true nearly all of the time (4). The scale is rated based on how the subject has felt over the past month. The total score ranges from 0–100, with higher scores reflecting greater resilience.

|  | Items | not true at all | rarely true | sometimes true | often true | true nearly all of the time |
| --- | --- | --- | --- | --- | --- | --- |
| 1 | I am able to adapt when changes occur. |  |  |  |  |  |
| 2 | I have one close and secure relationship. |  |  |  |  |  |
| 3 | Sometimes fate or God helps me. |  |  |  |  |  |
| 4 | I can deal with whatever comes my way. |  |  |  |  |  |
| 5 | Past successes give me confidence. |  |  |  |  |  |
| 6 | I try to see the humorous side of things when I am faced with problems. |  |  |  |  |  |
| 7 | Having to cope with stress can make me stronger. |  |  |  |  |  |
| 8 | I tend to bounce back after illness, injury or other hardships. |  |  |  |  |  |
| 9 | I believe most things happen for a reason. |  |  |  |  |  |
| 10 | I make my best effort, no matter what. |  |  |  |  |  |
| 11 | I believe I can achieve my goals, even if there are obstacles. |  |  |  |  |  |
| 12 | Even when hopeless, I do not give up. |  |  |  |  |  |
| 13 | In times of stress, I know where to find help. |  |  |  |  |  |
| 14 | Under pressure, I stay focused and think clearly. |  |  |  |  |  |
| 15 | I prefer to take the lead in problem-solving. |  |  |  |  |  |
| 16 | I am not easily discouraged by failure. |  |  |  |  |  |
| 17 | I think of myself as a strong person when dealing with life’s challenges and difficulties. |  |  |  |  |  |
| 18 | I make unpopular or difficult decisions. |  |  |  |  |  |
| 19 | I am able to handle unpleasant or painful feelings like sadness, fear, and anger. |  |  |  |  |  |
| 20 | I have to act on a hunch. |  |  |  |  |  |
| 21 | I have a strong sense of purpose in life. |  |  |  |  |  |
| 22 | I feel like I am in control. |  |  |  |  |  |
| 23 | I like challenges. |  |  |  |  |  |
| 24 | I work to attain goals. |  |  |  |  |  |
| 25 | I take pride in my achievements. |  |  |  |  |  |
